# Noninvasive Molecular Imaging of Disease Activity in Atherosclerosis

**DOI:** 10.1161/CIRCRESAHA.116.307971

**Published:** 2016-07-07

**Authors:** Marc R. Dweck, Elena Aikawa, David E. Newby, Jason M. Tarkin, James H.F. Rudd, Jagat Narula, Zahi A. Fayad

**Affiliations:** From the Translational and Molecular Imaging Institute (M.R.D., Z.A.F.) and Zena and Michael A. Wiener Cardiovascular Institute (M.R.D., J.N., Z.A.F.), Icahn School of Medicine at Mount Sinai, New York; Centre for Cardiovascular Science, University of Edinburgh, Edinburgh, United Kingdom (M.R.D., D.E.N.); Cardiovascular Division, Department of Medicine, Center for Excellence in Vascular Biology, Brigham and Women’s Hospital, Harvard Medical School, Boston, MA (E.A.); and Division of Cardiovascular Medicine, University of Cambridge, Cambridge, United Kingdom (J.M.T., J.H.F.R.).

**Keywords:** 18F-Fluorodeoxyglucose, atherosclerosis, disease progression, inflammation, myocardial infarction

## Abstract

Major focus has been placed on the identification of vulnerable plaques as a means of improving the prediction of myocardial infarction. However, this strategy has recently been questioned on the basis that the majority of these individual coronary lesions do not in fact go on to cause clinical events. Attention is, therefore, shifting to alternative imaging modalities that might provide a more complete pan-coronary assessment of the atherosclerotic disease process. These include markers of disease activity with the potential to discriminate between patients with stable burnt-out disease that is no longer metabolically active and those with active atheroma, faster disease progression, and increased risk of infarction. This review will examine how novel molecular imaging approaches can provide such assessments, focusing on inflammation and microcalcification activity, the importance of these processes to coronary atherosclerosis, and the advantages and challenges posed by these techniques.

The past 15 years have witnessed a remarkable expansion in noninvasive cardiovascular imaging technology. Indeed, we now have access to a wide spectrum of modalities, each offering distinct and potentially complementary information with respect to the pathophysiology of atherosclerosis. Considerable interest has surrounded the use of this technology to improve cardiovascular risk prediction so that we can better identify high-risk patients, allowing us to intervene and avert subsequent myocardial infarction. For many years, the major focus in atherosclerosis imaging research has been to identify individual coronary plaques at high risk of rupture, the so-called vulnerable plaque. Ultimately, this strategy has failed to have a major impact on clinical care, prompting many to consider a switch of emphasis to pan-coronary and pan-vascular assessments of disease activity that may be more closely related to the *v*ulnerable patient: those subjects at highest risk of cardiovascular events.^1^ This review will describe novel noninvasive imaging strategies developed to tackle this issue; in particular, how the measures of disease activity targeted to both inflammation and microcalcification might be used to identify patients at highest risk of stroke and myocardial infarction.

## Problems With the Vulnerable Plaque Paradigm

**Figure 1.**
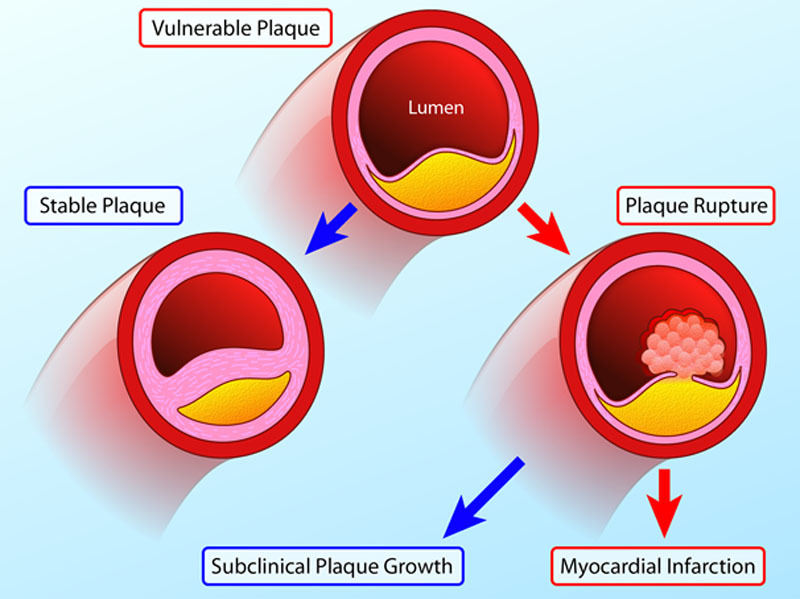
**Natural history of the vulnerable plaque**. Vulnerable atherosclerotic plaques are thought to account for the majority of myocardial infarctions and are characterized by macrophage inflammation, a thin fibrous cap, positive remodeling, microcalcification, and angiogenesis. Histological and imaging studies conducted post myocardial infarction (MI) have consistently associated these plaques with rupture and myocardial infarction. However, in prospective observational studies, only a minority of these plaques go on to cause adverse clinical events (red arrows). This is because many vulnerable plaques will in fact heal and stabilize via multiple processes, including calcification. Although a proportion will go on to rupture, the majority of such events remain subclinical resulting in plaque growth rather than MI. As a consequence, the number of vulnerable plaques seems to greatly outnumber the clinical events that ensue (Illustration credit: Ben Smith).

The majority of acute coronary events are because of atherosclerotic plaque rupture. However, identifying plaques at risk of rupture, the so-called vulnerable plaque, has proved problematic. The majority of plaques causing myocardial infarction are nonobstructive on antecedent coronary angiography,^2,3^ and up to one third of ruptured plaques demonstrate <75% cross-sectional vascular area narrowing at postmortem.^3^ These lesions are, therefore, frequently missed on angiography and stress testing, prompting interest in novel imaging strategies that might better predict myocardial infarction. Histological studies have consistently associated several key adverse plaque characteristics with rupture and myocardial infarction. These include a thin fibrous cap, macrophage infiltration, a large necrotic core and plaque volume, microcalcification, angiogenesis, and intraplaque hemorrhage. These features are often observed in constellation in lesions known as thin-capped fibroatheroma, with each feature representing a potential imaging target for identifying plaque vulnerability regardless of the extent of luminal stenosis. Indeed, this has been the subject of intense research over the past 10 to 15 years and the principle underlying the development of numerous invasive and noninvasive imaging techniques (Figure 1). However, to date this approach has failed to affect the clinical practice.

The PROSPECT trial (A Prospective Natural-History Study of Coronary Atherosclerosis) investigated whether the detection of thin-capped fibroatheroma by virtual histology intravascular ultrasound would predict adverse clinical events.^4^ In this study, 595 virtual histology intravascular ultrasound–defined thin-capped fibroatheromas were identified in 697 patients; however, after a median follow-up of 3.4 years, only 6 lesions resulted in myocardial infarction. Similarly, the VIVA study (VH-IVUS in Vulnerable Atherosclerosis) identified 550 virtual histology–thin-capped fibroatheromas with only 8 resulting in a major adverse cardiovascular event not related to stent restenosis.^5^ Indeed, the low predictive value of individual plaques progressing to cause events has emerged as the major limitation with the vulnerable plaque strategy.^6,7^ Although imaging and histological studies conducted post myocardial infarction have demonstrated that the above adverse characteristics have consistently been associated with culprit and ruptured plaques, prospective observational studies have shown that such plaques are, in fact, relatively common but go on to cause myocardial infarction in only a small minority of cases.^8^

Why then do vulnerable plaques outnumber the cardiac events that they cause? The answer lies in the natural history of these lesions (Figure 1). First, the majority of these inflamed high-risk lesions are likely to heal and stabilize with time rather than rupture. In particular, progressive calcification sees the transition from the early stages of high-risk microcalcification to the stable end stages of macroscopic calcification.^9^ Second, even in those lesions where the healing process is unsuccessful and plaque rupture occurs, the majority of these events seem to be subclinical, resulting in silent plaque growth, rather than myocardial infarction.^8,10^ Indeed, evidence of old healed plaque rupture is seen in more than four fifths of lesions with >50% luminal stenosis.^10^ As a consequence, although retrospective studies demonstrate that vulnerable plaques are consistently responsible for myocardial infarction, prospective studies indicate that only a minority of these supposedly high-risk plaques proceed to cause clinically apparent events. The worth of identifying vulnerable plaques has, therefore, been questioned indeed if the majority go on to cause no harm, how can their treatment be justified?^8,11^

## Targeting the Vulnerable Patient

Strategies focused on broader patient-related factors have proved more effective. Cardiovascular risk scores, such as the Framingham risk score (FRS), have been used for several decades to estimate a patient’s risk of having a cardiovascular event based on well-established epidemiological studies and the presence of cardiovascular risk factors, such as age, diabetes mellitus, hypertension, and smoking. Although useful on a population level, the accuracy of predicting a patient’s individual risk is limited. Interest has, therefore, surrounded noninvasive imaging techniques that can directly image the disease process in a patient’s coronary arteries and, therefore, provide more personalized estimates of risk.

The atherosclerotic plaque burden can be measured using several simple and inexpensive imaging approaches. These predict adverse cardiovascular events presumably on the basis that the more plaques a patient has, the more likely one will rupture or erode and cause an event. Computed tomographic (CT) calcium scoring quantifies the amount of macroscopic calcification in the coronary arteries. This provides a surrogate of the total coronary atherosclerotic plaque burden and improves risk prediction models when added to the Framingham risk scores.^12–14^ More detailed plaque assessments are now available with coronary CT angiography, allowing visualization of both calcified and noncalcified plaque, as well as adverse plaque characteristics.^6,15^ Yet, despite these considerable technological advances, individual risk prediction remains limited.

## Molecular Imaging of Disease Activity

A limitation of anatomic plaque assessments is that although they provide an indication as to how much plaque has accumulated over time, they give no indication as to the current activity of the disease process. They are, therefore, unable to distinguish those patients who have burnt-out, stable disease that is no longer metabolically active versus those with active atheroma. This is of potential importance because patients with active disease will develop multiple unstable plaques over time; and although the majority will heal without incident, overall there will be an increased probability that one will rupture when the blood is thrombogenic and cause an event. The poor prognosis associated with an active disease process was first suggested by studies investigating disease progression. Raggi et al^16^ demonstrated the added value of examining the progression in plaque burden compared with one-off baseline measures. In a cohort of patients treated with statin therapy who underwent serial CT calcium scoring those who demonstrated <15% annual progression in their CT calcium score had an excellent prognosis with few events irrespective of their baseline plaque burden. By contrast, subjects who demonstrated >15% change in their calcium score had an adverse prognosis, which increased progressively depending on their baseline disease burden. Although >10-year old, this study was consistent with the hypothesis that subjects with increased disease activity and greater rates of progression are more likely to have events.

Changes in the CT calcium score provide only an indirect measure of disease activity and require serial imaging. Improved clinical imaging methods are, therefore, required to measure disease activity directly. The emergence of molecular imaging now allows us to do exactly that. Molecular tracers targeted to pathological processes of interest are injected into the body, where they accumulate at sites of increased disease activity. These tracers are labeled with an imaging reporter that provides signal on the relevant imaging platform. Potentially, the activity of any disease process can be assessed depending on the availability of a suitable imaging tracer and scanner. However in practice, the strict requirements of the US Food and Drug Administration (FDA) for regulatory approval make the development of novel clinical tracers both time consuming and expensive. Human research has, therefore, largely focused on tracers already approved for noncardiac conditions but which target disease processes relevant to atherosclerosis and acute plaque rupture. In particular, markers of inflammation and microcalcification activity have been evaluated. These are discussed in detail here, although an array of other tracers targeting processes such as angiogenesis, hypoxia, and plaque hemorrhage are also in development.^17–19^

## Ultrasmall Particles of Iron Oxide Imaging of Inflammation

**Figure 2.**
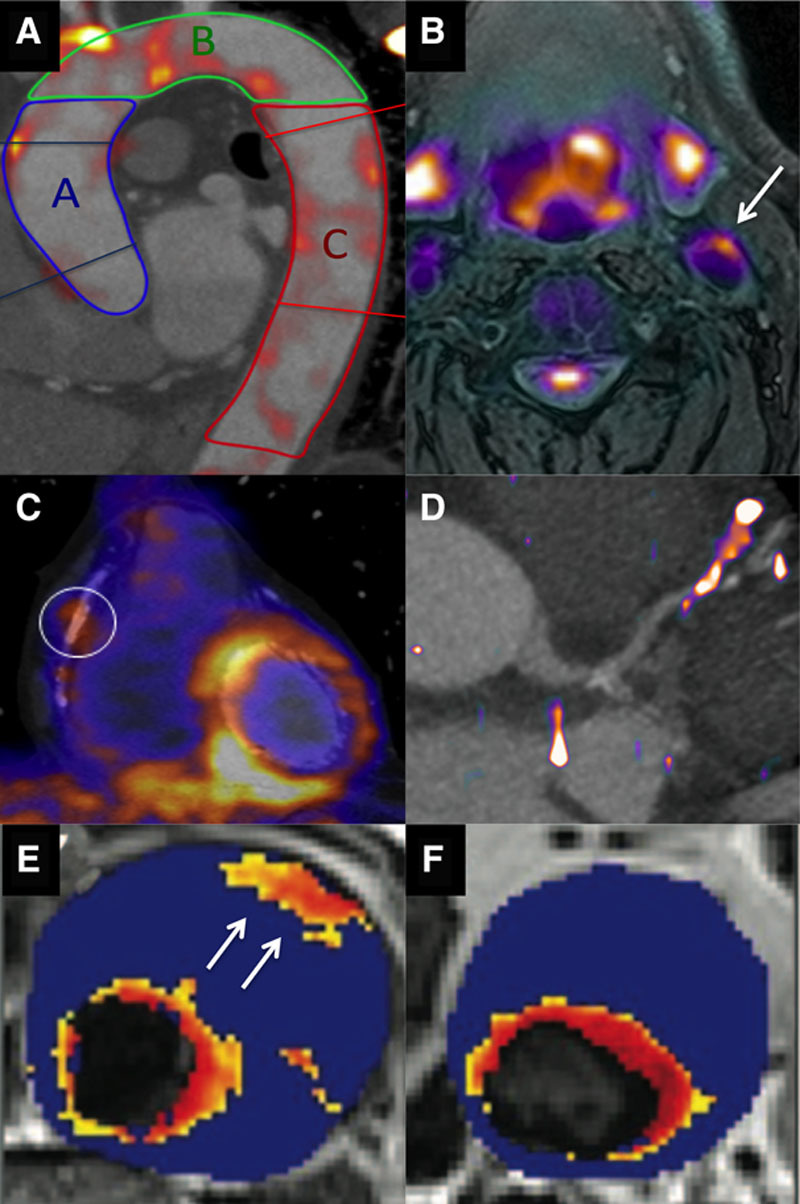
**Molecular imaging of vascular inflammation activity**. **A**, 18F-fluorodeoxyglucose (18F-FDG) positron emission tomography (PET) image fused with contrast computed tomographic (CT) angiogram of the thoracic aorta, demonstrating regions of increased activity in the ascending aorta (blue), aortic arch (green), and descending aorta (red). **B**, 18F-FDG PET/magnetic resonance (MR) image of the carotid arteries demonstrating increased activity in the left carotid artery and the excellent soft tissue contrast provided by MR. **C**, 18-FDG PET fused with a CT coronary angiogram demonstrating increased uptake in the left ventricle but also in a remote plaque in the mid right coronary artery. Image in panel C reproduced from Cheng et al^21^ with permission of the publisher. Copyright © 2012, the Society of Nuclear Medicine and Molecular Imaging, Inc. **D**, 68Ga-DOTATATE PET/CT image with increased activity localizing to a plaque in the mid left anterior descending artery. **E**, T2* Map from patients with an abdominal aortic aneurysm that had been administered ultra small particles of iron oxide (USPIO). A hot spot is observed in the anterior wall of the aneurysm (arrow). **F**, In a second patient, focal areas of increased USPIO uptake can be observed (the increased signal adjacent to the lumen is considered normal because of high signal in the blood pool). Images in panels E and F reproduced from Richards et al^23^ with permission of the publisher. Copyright © 2011, Wolters Kluwer Health, Inc.

Macrophages play a pivotal role in the destabilization of atherosclerotic plaques, secreting fibrous cap–degrading matrix metalloproteinases, proinflammatory cytokines, and prothrombotic tissue factor.^20^ These then weaken the fibrous cap and drive growth of the necrotic core, increasing the propensity to plaque rupture and clinical events. Multiple molecular imaging techniques have been developed to measure inflammatory activity in atherosclerosis (Figure 2).

Macrophages can be imaged directly using iron oxide contrast agents that have superparamagnetic properties on T2*-weighted sequences. Iron oxide is clinically approved for the treatment of iron deficiency anemia, and although concerns have been expressed about the risk of hypersensitivity reactions leading to restricted availability in the United States, these seem less of an issue at the lower doses used in imaging. After injection of ultrasmall superparamagnetic particles of iron oxide (USPIO), these particles are removed from the circulation by the reticuloendothelial system and accumulate in macrophages present in atherosclerotic plaques. Iron oxide causes distortion in the magnetic resonance signal, which can be used to localize USPIO accumulation and to estimate the macrophage burden in a range of disease processes, including atherosclerosis, abdominal aortic aneurysms, and myocardial infarction.^22–24^

Kooi et al^25^ studied 11 symptomatic patients scheduled for carotid endarterectomy with USPIO-enhanced magnetic resonance, and they found a 24% decrease in signal intensity in the culprit vessel on T2*-weighted sequences, with histological evidence of USPIO uptake in 75% of these plaques post surgery. The USPIO signal also seems to be modifiable with drug therapy and, therefore, may be of value in assessing the anti-inflammatory properties of atherosclerotic therapies. Tang et al^26^ showed that the intensity of the USPIO signal could be reduced by 3 months of statin therapy, and that this reduction was increased with high- versus low-dose statin. Current limitations of USPIO-enhanced magnetic resonance imaging with agents such as ferumoxtran are the need for 2 scans, one before USPIO administration and another one ≈36 hours after injection.^27^ Accurate coregistration is then required to detect differences in the T2* signal because of USPIO accumulation. This can be challenging in complex 3-dimensional vascular structures. Moreover, T2* artifact and high blood pool activity are frequently observed, making the detection of true signal in atherosclerotic plaques difficult. Although these issues can largely be overcome in the carotids and aorta, they currently preclude imaging in the small and constantly moving coronary arteries.

## Positron Emission Tomography Imaging of Inflammation Activity

Positron emission tomography (PET) is a nuclear imaging modality used for a variety of clinical purposes. It is ideally suited to measure disease activity, using radiolabeled tracers targeted to specific pathological processes. PET tracers combine a positron emitter (usually 18F because of its advantageous half-life) with a molecular vehicle targeting the cellular process of interest. After injection, these tracers accumulate in areas of disease activity, emitting positrons, which collide with nearby electrons. This collision results in particle annihilation and the release of a pair of photons with a specific energy. Simultaneous detection of these paired photons allows the PET scanner to localize tracer accumulation with exquisite sensitivity and a spatial resolution of ≈4 mm. Two tracers, in particular, hold promise in assessing atherosclerosis: 18F-fluorodeoxyglucose (18F-FDG) as a marker of inflammation activity^28^ and 18F-fluoride as a marker of newly forming microcalcification.^29^ These tracers are used for the routine clinical imaging of oncology patients. They are, therefore, FDA approved, commercially available, inexpensive, and have established excellent safety records.

## 18F-FDG PET

18F-FDG is a PET tracer and glucose analog that is taken up by metabolically active cells. On this basis, it has become widely used to image cancer but more recently has been used as a marker of vascular inflammation given that macrophages use more glucose than surrounding cells, particularly in hypoxic conditions.^30^

18F-FDG PET has been most extensively investigated in the carotid arteries and aorta (Figure 2) with uptake correlating with macrophage burden in excised carotid plaques^31^ and multiple adverse imaging plaque characteristics.^32,33^ Importantly, accumulating data demonstrate the prognostic value of 18F-FDG PET, albeit largely in the form of single-center, retrospective studies. In the Dublin Carotid Atherosclerosis Stroke Study, carotid artery inflammation detected by 18F-FDG was useful for identifying patients most at risk of early stroke recurrence.^34^ In another retrospective study of 513 patients without cancer, inflammatory disease or previous cardiovascular disease but who underwent PET imaging for oncological evaluation, the aortic 18F-FDG PET signal again provided independent prediction of cardiovascular events after a median follow-up of 4.2 years (hazard ratio, 4.71, *P*<0.001).^35^ Similarly, in 1089 asymptomatic adults being screened for cancer increased carotid 18F-FDG uptake predicted future stroke with incremental value above FRS.^36^

Scan–rescan reproducibility of the vascular 18F-FDG signal is excellent^37^ and coupled with the established mechanistic and prognostic data available for this tracer; 18F-FDG PET is increasingly being used as an end point in trials of novel atherosclerosis therapies. The excellent reproducibility means that such studies require relatively few patients (n=30–50) to demonstrate an anti-inflammatory treatment effect, which can be observed quickly after drug initiation (3–6 months). Vascular 18F-FDG studies are, therefore, relatively low cost and time efficient, serving as an economical introduction to larger and more expensive clinical end point trials. The beneficial effects of statin therapy are well documented, so it is reassuring that statins have demonstrated a consistent and clear reduction in the vascular 18F-FDG PET signal, proportional to the dose of statin used.^38–40^ In contrast, inhibitors of lipoprotein-associated phospholipase A_2_, failed to modify both the vascular 18F-FDG signal and the hard clinical end points.^41,42^ Similarly, dalcetrapib, a cholesteryl ester transfer protein inhibitor, demonstrated no impact on either vascular 18F-FDG activity nor clinical events.^43,44^

Translation of 18F-FDG imaging in to the coronary arteries has proved more challenging. This is because glucose is the predominant energy source of the myocardium, such that avid uptake of 18F-FDG by the left ventricle is frequently observed. Although myocardial 18F-FDG uptake can be suppressed by dietary restrictions (that aim to switch myocardial metabolism from glucose to free fatty acids), this is ineffective in 20% to 30% of cases. Indeed in a recent study, >50% of the coronary territories assessed were not interpretable because of spill over of myocardial 18F-FDG uptake.^45^ Until improved methods of myocardial suppression are developed, this is likely to limit the assessment of 18F-FDG uptake in individual coronary plaques. It may, however, be possible to measure more generalized 18F-FDG uptake in the proximal coronary vessels or the ascending aorta. Rogers et al^46^ studied post-acute coronary syndrome in 10 patients, demonstrating the increased uptake in the left main stem and ascending aorta compared with 15 patients with stable angina. Similarly, Joshi et al^47^ demonstrated increased 18F-FDG uptake in the thoracic aorta of 40 patients after myocardial infarction compared with 40 stable patients, despite similar plaque burden. Whether this increased vascular inflammation activity represents the cause or effect of the acute infarct remains to be established, although a correlation was observed between infarct size and aortic 18F-FDG uptake,^47^ consistent with the latter interpretation and recent preclinical data.^48^

## Alternative PET tracers

The fact that reliable coronary evaluation with 18F-FDG is often not possible because of high myocardial muscle uptake, coupled with its inherent lack of cellular specificity, drives the search for alternative PET tracers targeting atherosclerotic inflammation. 68Ga-DOTATATE ([1,4,7,10-tetraazacyclododecane-*N*,*N*′,*N*″,*N*′″-tetraacetic acid]-d-Phe(1),Tyr(3)-octreotate) is a PET tracer with high specific binding affinity for the somatostatin receptor subtype-2 (≈0.2 nmol/L), which is currently used clinically for neuroendocrine tumor imaging. Upregulation of the somatostatin receptor subtype-2 seems to occur on the cell surface of activated macrophages, offering a potential novel imaging target for tracking vascular inflammation. Initial studies are encouraging, and there is some histological evidence in mice showing significant correlation between the aortic 68Ga-DOTATATE PET signal and macrophage density (%CD68 staining).^49^ In a retrospective study, Rominger et al^50^ observed detectable 68Ga-DOTATATE uptake in the left anterior descending artery in each of the 70 patients evaluated, with increased tracer activity seen among patients with coronary calcification or a history of cardiovascular events. In another retrospective study, increased vascular 68Ga-DOTATATE uptake was, again, observed in the aorta, carotid and iliac arteries in patients with cardiovascular risk factors and coronary calcification.^51^ Although low myocardial binding of 68Ga-DOTATATE is advantageous for coronary imaging, high physiological liver uptake can potentially obscure interpretation of signals originating for the distal right coronary artery. The use of this tracer for atherosclerosis imaging is being studied in more detail in the ongoing prospective Vascular Inflammation Imaging Using Somatostatin Receptor Positron Emission Tomography (VISION) study (ClinicalTrial.gov NCT02021188).

11C-PK11195 is one of the several PET tracers targeting the translocator protein, a receptor that is highly expressed on the surface of macrophages. This family of tracers has, therefore, also been used to target vascular inflammation.^52,53^ Fujimura et al^54^ showed specific binding of 3H-PK11195 to macrophages in human carotid atheroma, although Pugliese et al^55^ demonstrated increased 11C-PK11195 PET activity in patients with symptomatic large-vessel vasculitis. Gaemperli et al^53^ demonstrated increased activity of 11C-PK11195 in culprit carotid plaques poststroke with uptake localizing to macrophages and translocator protein expression on histology. However, using 11C in clinical studies is challenging because of its half-life, so that 18F-labeled translocator protein tracers are keenly anticipated, although issues also surround genetic polymorphisms that seem to have a major effect on tracer-binding affinity.

Finally, PET imaging with 18F-fluorodeoxymannose has recently been described in a preclinical study.^56^ Mannose is an isomer of glucose, and the mannose receptor is upregulated on alternatively activated or M2 macrophages in high-risk plaque. There is, therefore, hope that 18F-fluorodeoxymannose PET may provide improved detection of such lesions. Macrophages demonstrate a 35% increase in tracer uptake compared with 18-FDG, with a similar pattern of vascular uptake in a rabbit model of atherosclerosis.^56^ Further studies are awaited, although they may be limited by the difficulties in radiolabeling mannose.

## The Link Between Inflammation and Calcification

The exact role of calcification in atherosclerosis remains to be defined. Although significant progress in our understanding has been achieved over recent years, many of the proposed mechanisms described below requires confirmation by future studies.

**Figure 3.**
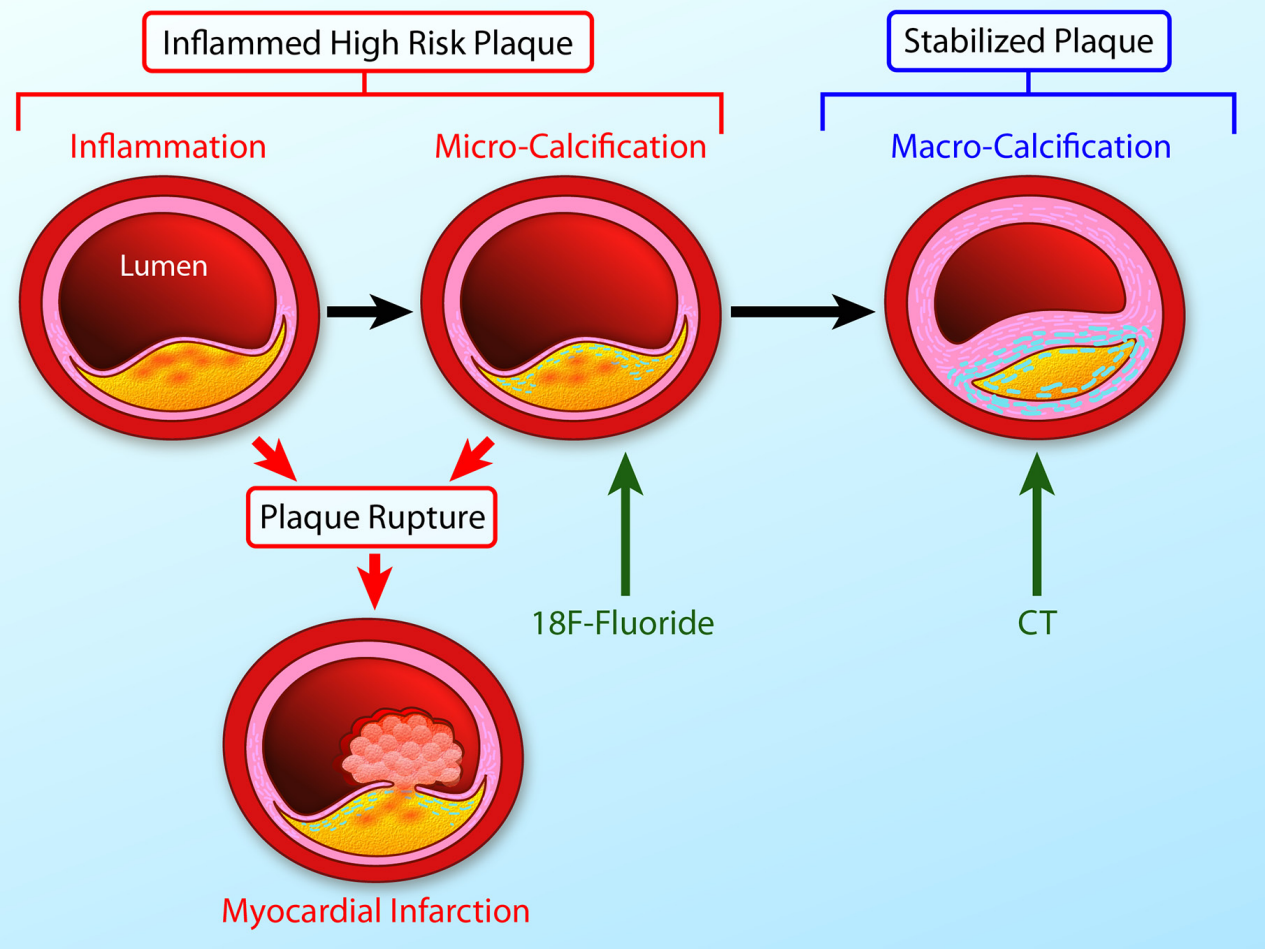
**The link between inflammation, microcalcification, and macrocalcification**. A large necrotic core, a thin fibrous cap, and an intense inflammation are key precipitants of acute plaque rupture and myocardial infarction. Intimal calcification is thought to occur as a healing response to this intense necrotic inflammation. However, the early stages of microcalcification (detected by 18F-fluoride positron emission tomography) are conversely associated with an increased risk of rupture. In part, this is because of residual plaque inflammation and in part because microcalcification itself increases mechanical stress in the fibrous cap further increasing propensity to rupture. With progressive calcification, plaque inflammation becomes pacified and the necrotic core walled off from the blood pool. The latter stages of macrocalcification (detected by computed tomographic) are, therefore, associated with plaque stability and a lower risk of that plaque rupturing (Illustration credit: Ben Smith).

Emerging evidence suggests that intimal calcification occurs as a healing response to intense inflammation and cell death within the atherosclerotic plaque, mediated by extracellular vesicles (Figure 3). It seems to be a different disease process to the medial calcification often unassociated with inflammation and more commonly observed in the larger arteries of patients with renal failure and altered calcium metabolism (eg, patients on dialysis and osteoporosis).^57^ In the coronary arteries, intimal calcification predominates. Using longitudinal molecular imaging studies, Aikawa et al^58^ linked inflammation and intimal calcification in a mouse model of atherosclerosis, demonstrating a close association between the early stages of in vivo microcalcification and macrophage inflammatory activity (*R*^2^=0.93). The link between inflammation and calcification also seems closely related to cell death within the plaque and the release of extracellular vesicles.^59–61^ These pathological extracellular vesicles are loaded with pro-osteogenic mediators (eg, proteins and micro-RNAs), containing high concentrations of calcium and phosphate, and seem to act as nucleation sites for the formation of hydroxyapatite crystal.^9,62–64^ Extracellular vesicles, recently demonstrated to be of exosomal origin, are released directly by inflammatory macrophages^65^ and by vascular smooth muscle cells, implying that atherosclerotic plaques contain a continuous source of the precursors to microcalcification.^9,64^ Concurrent with this process, a range of resident vascular and recruited progenitor cells differentiate into those with an osteoblastic or chondrocytic phenotypes. This differentiation process seems to be associated with inflammation, promoted by proinflammatory cytokines, particularly tumor necrosis factor-α.^59^ These procalcific cells then provide an alternative source of calcifying extracellular vesicles, driving the ongoing formation of microcalcifications until large stable sheets of macroscopic calcification develop.

## Differences Between Microcalcification and Macrocalcification

The presence of calcium in the coronary arteries can have diverse clinical implications. It is useful to consider calcification as a 2-phase process: the initial stage of microcalcification and the end stages of macroscopic calcium formation (Figure 3).^66^ Macroscopic calcific deposits are traditionally associated with plaque stability, supported by recent models of mechanical stress.^67^ Indeed, we consider them to represent the end stage of the healing process, effectively walling off the inflamed plaque contents in a process similar to other intense inflammatory conditions, such as tuberculosis. These large deposits are readily identified by CT. Indeed, CT calcium scoring is a powerful predictor of coronary events.^68^ Of note, extensively calcified plaques themselves only rarely result in rupture and adverse events, but rather calcium scoring acts as a biomarker of overall disease and coronary plaque burden.

By comparison, microcalcification represents the early stages of intimal calcium formation and is associated with high risk and culprit atherosclerotic plaque in histopathologic and imaging studies alike.^69–73^ This may, in part, reflect the close relationship between the microcalcification and the inflammation thought to act as its trigger.^58^ However, recent data have suggested that microcalcification might itself directly contribute to plaque rupture, greatly amplifying mechanical stresses on the surface of the fibrous plaque.^74,75^ In particular, deposits ranging between 5 and 60 µm are associated with an increase in local stresses >500%.^75,76^ Whether directly or indirectly, microcalcification, therefore, seems closely related to the processes driving plaque rupture and although it remains beyond the resolution of CT, these structures can now be detected noninvasively using molecular imaging.

## PET Imaging of Microcalcification Activity

18F-fluoride has been used safely as a PET bone tracer for over 40 years but has only recently been assessed in the vasculature. Indeed, the first description was in 2010 by Derlin et al^77^ who retrospectively studied patients who had been scanned for investigation of cancer. They described increased uptake in the aorta, carotids and femoral vessels in 57 of the 75 patients studied. Although sites of 18F-fluoride uptake were commonly observed in close association with regions of existing calcium on CT, increased uptake was also observed in their absence. Moreover, the vast majority of calcific lesions on CT did not demonstrate increased 18F-fluoride uptake, suggesting that the 2 approaches provide different information about vascular calcification. The same group went on to establish an association between femoral 18F-fluoride uptake and cardiovascular risk factors^78^ and to demonstrate that this tracer provides distinct information to 18F-FDG.^79^

**Figure 4.**
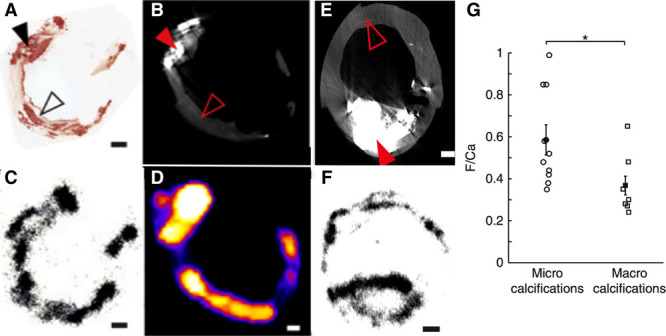
**18F-Fluoride preferentially binds microcalcification beyond the resolution of computed tomography (CT**). Images are taken ex vivo of a carotid endarterectomy specimen excised from a patient who had a recent stroke. **A**, Histological section of the excised plaque stained for calcium with Alizarin red. **B**, Filled black arrow shows an area of dense macroscopic calcification that is visible on micro-CT. By comparison, the empty black arrow head demonstrates areas of microcalcification that are beyond the resolution of the micro-CT but by comparison demonstrate avid binding with 18F-fluoride on both autoradiography (**C**) and micro–positron emission tomography imaging (**D**). **E**, A second carotid endarterectomy sample from a patient post stroke demonstrates a large macro calcific deposit on micro-CT. **F**, Autoradiography shows that although 18F-fluoride is able to bind to the surface of the plaque, it is unable to penetrate in to the center. As a consequence of this effect, 18-fluoride binds preferentially to regions of microcalcification compared with macroscopic deposits. Reprinted from Irkle et al^29^ with permission. Copyright © 2015, Macmillan Publishers Limited.

Although these studies provided early insight, the exact mechanisms underlying 18F-fluoride uptake in the vasculature have only recently been elucidated. Irkle et al^29^ provided comprehensive assessment of carotid endarterectomy samples using electron microscopy, autoradiography, histology, and preclinical and clinical PET/CT to analyze 18F-fluoride binding. They demonstrated that 18F-fluoride adsorbs to calcified deposits within plaque with high affinity and is both selective and specific. Moreover, 18F-fluoride was able to distinguish between areas of macro- and microcalcification, binding preferentially to the latter. It, therefore, provided differential information to CT, which was only able to detect large macroscopic deposits. Indeed, 18F-fluoride is the only currently available clinical imaging platform that can noninvasively detect microcalcification in active unstable atheroma. The explanation for 18F-fluoride’s preferential binding to microcalcification is the high surface area of hydroxyapatite in these nanocrystalline areas. By comparison, in large macroscopic deposits, much of the hydroxyapatite is internalized and not available for binding so that 18F-fluoride uptake is only observed at the periphery (Figure 4).

Behesti et al^80^ first described 18F-fluoride activity in the heart, with further studies localizing this activity to the valves of patients with aortic stenosis^81,82^ and to individual coronary plaques.^83^ In aortic stenosis, isolated 18F-fluoride uptake predicts the location of future macroscopic calcium formation on CT, accurately predicting progression of the aortic valve CT calcium score,^84,85^ and correlating with alkaline phosphatase activity on histology.^85^ 18F-fluoride, therefore, acts as a marker of vascular calcification activity in aortic stenosis, appearing to bind to newly forming microcalcification within the valve leaflets.^86^

**Figure 5.**
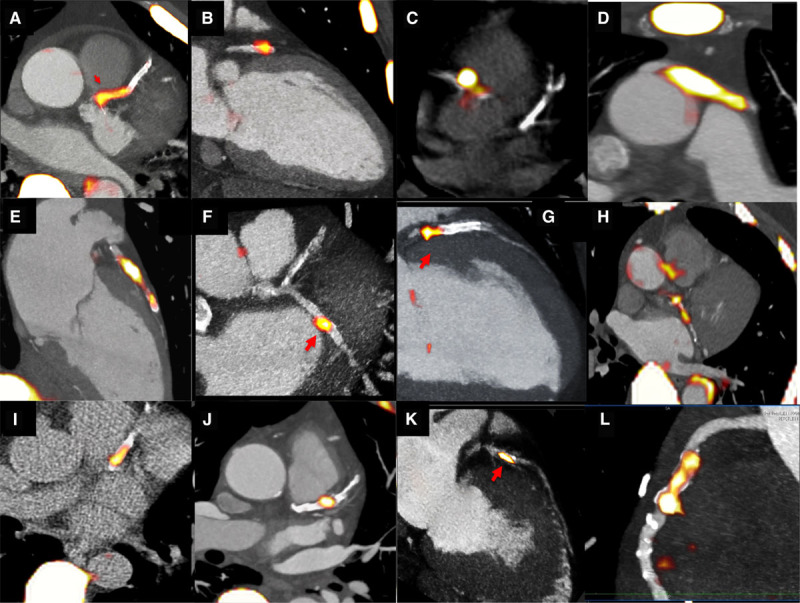
**18F-fluoride PET imaging in the coronary arteries**. Multiple examples of 18F-fluoride localizing to individual coronary plaques on fused positron emission tomography/computed tomography images of the heart. This can be observed in the left anterior descending artery (**A, B, E, G, I, J**, and **K**), circumflex (**F** and **H**) and right coronary arteries (**C** and **L**), and in a saphenous vein graft (**D**).

In the coronary arteries, 18F-fluoride is the first tracer to localize to individual coronary plaques with little background activity and excellent signal-to-noise ratio (Figure 5).^83^ Those plaques demonstrating increased 18F-fluoride uptake have multiple high-risk characteristics when assessed with virtual histology intravascular ultrasound, including microcalcification, positive remodeling, and a large necrotic core, findings confirmed in pathological validation studies of the carotid arteries.^45^ 18F-fluoride also seems to localize to ruptured culprit plaque post myocardial infarction. In a study of 40 patients who had a recent myocardial infarction, 37 demonstrated increased uptake in their adjudicated culprit plaque.^45^ This would suggest that microcalcification is indeed implicated in atherosclerotic plaque rupture, although it remains impossible to know whether increased 18F-fluoride activity was truly present before the rupture event or whether it developed in response. Moreover for the reasons discussed above, it would seem likely that in observational cohort studies, the majority of 18F-fluoride–positive plaques would heal, rather than causing events. Nevertheless 18F-fluoride holds promise in improving our understanding of the pathophysiology of atherosclerosis and in predicting events at the patient level. Indeed, the ability of hybrid 18F-fluoride PET/CT to predict disease progression and to identify patients at risk of myocardial infarction is currently being tested in a large multicenter and prospective observational study, PREFFIR (Prediction of Recurrent Events With 18F-Fluoride; ClinicalTrial.gov NCT02278211).

## Hybrid Coronary Imaging

Given the highly complex, multifaceted pathophysiology underlying atherosclerosis and its progression to plaque rupture and myocardial infarction, accurate risk prediction may well depend on a similarly multifaceted imaging approach, simultaneously assessing a range of pathophysiological and prognostic factors. Hybrid cardiovascular imaging has the potential to provide exactly that information. PET/CT scanners incorporate both CT and PET within the same gantry allowing sequential imaging with the patient in the same position. The 2 images can then be fused and used to provide complimentary information: anatomy from the CT and disease activity from PET. Progressively, more complex imaging protocols have been introduced combining CT calcium scoring, contrast CT angiography, and PET motion correction so that a single scan can potentially provide an assessment of plaque burden, high-risk plaque characteristics, luminal obstruction, and disease activity. The hope is that the combined predictive capability of these parameters will facilitate highly accurate prognostic models, allowing systemic therapies to be targeted to patients at highest risk and reducing the incidence of future myocardial infarction (Figure 4). Again this combined strategy will be assessed in PREFFIR.

## Optimization of Coronary PET Imaging

Further work is required to optimize 18F-fluoride imaging in the coronary arteries. This is made particularly difficult by both the effects of coronary motion and the small caliber of these vessels, causing partial voluming.^87^ ECG gating improves localization of the signal to the coronary arteries but discards 75% of the data increasing noise.^45^ New image analysis techniques model and then correct for both cardiac and respiratory motion without discarding any data, allowing improved localization, increased signal and reduce noise.^88^ Background 18F-fluoride uptake in the coronary arteries seems lower than in the blood pool of the right atrium because of the partial voluming and the influence of low uptake in the adjacent lungs and myocardium. Further work is required to account for this issue and to determine the best method for quantifying coronary 18F-fluoride uptake and whether increased activity is present. For example, it remains unclear if the correcting coronary activity for background uptake in the blood pool or adjacent coronary vasculature is preferable. Moreover, although 18F-fluoride preferentially binds newly developing areas of microcalcification, there remains some binding to the surface of large macroscopic deposits, suggesting that coronary uptake should perhaps also account for the underlying CT calcium score. The scan–rescan reproducibility of these image analysis approaches also needs to be established before studies examining how the coronary 18F-fluoride signal changes with time are performed. Finally, a potential barrier to the future application of PET/CT is the associated high-radiation exposure (≈10 mSV). This is likely to preclude serial imaging of patients and limit our ability to track disease progression and response to therapy. Although CT radiation doses are being reduced rapidly, an alternative approach is to use PET/magnetic resonance imaging that might not only reduce radiation by ≤70% but also improve motion correction and avoid the need for contrast administration.^89,90^

## Conclusions

Concerns have emerged with imaging strategies aimed at identifying individual vulnerable plaques in the coronary arteries. There is hope that newer strategies providing more global assessments of disease activity across the vasculature will lead to improved risk prediction, differentiating patients with truly stable disease compared with those with active potentially unstable atheroma. These strategies have largely focused on imaging inflammation in larger arteries and more recently microcalcification in the coronary arteries providing important pathophysiological insight and with the potential to ultimately influence patient management. However, these relatively expensive techniques will need to demonstrate both added value to risk instruments based on soluble biomarkers and traditional risk factors and ultimately that their use is clinically effective in reducing cardiovascular events within rigorous large-scale clinical trials.

## Sources of Funding

M.R. Dweck and D.E. Newby are supported by the British Heart Foundation (CH/09/002 to D.E. Newby and FS/14/78/31020 to M.R. Dweck). M.R. Dweck is the recipient of the Sir Jules Thorn Biomedical Research Award 2015 (M.R. Dweck). E. Aikawa research is supported by R01HL 114805 and R01HL 109506. J.H.F. Rudd is partly supported by the National Institute for Health Research Cambridge Biomedical Research Centre, the British Heart Foundation, and the Wellcome Trust. D.E. Newby is the recipient of a Wellcome Trust Senior Investigator Award (WT103782AIA) and a National Institute of Health Research Efficacy and Mechanism Evaluation Award (11/20/03). Z.A. Fayad is supported by the following grants: NIH/NHLBI R01 HL071021, NHLBI R01 HL128056, and NIH/NBIB R01 EB009638 and AHA14SFRN20780005.

## Disclosures

None.
